# Blood eosinophil related to maternal allergic rhinitis is associated with the incidence of allergic rhinitis in offspring: COCOA study

**DOI:** 10.1186/s12887-023-04156-1

**Published:** 2023-07-06

**Authors:** Eun-A Choi, Geumkyung Nah, Woo-Sung Chang, So-Yeon Lee, Dong In Suh, Kyung Won Kim, Youn Ho Shin, Kangmo Ahn, Soo-Jong Hong, Young Youl Kim, Hye-Ja Lee

**Affiliations:** 1 Division of Allergy and Respiratory Disease Research, Department of Chronic Disease Convergence Research, Korea National Institute of Health, Korea Disease Control and Prevention Agency, Cheongju, Republic of Korea; 2 Department of Pediatrics, Childhood Asthma Atopy Center, Humidifier Disinfectant Health Center, Asan Medical Center, University of Ulsan College of Medicine, Seoul, Republic of Korea; 3 Department of Pediatrics, Seoul National University College of Medicine, Seoul, Republic of Korea; 4 Department of Pediatrics, Yonsei University College of Medicine, Seoul, Republic of Korea; 5 Department of Pediatrics, CHA Gangnam Medical Center, CHA University College of Medicine, Seoul, Republic of Korea; 6 Department of Pediatrics, Environmental Health Center for Atopic Disease, Samsung Medical Center, Sungkyunkwan University School of Medicine, Seoul, Republic of Korea

**Keywords:** Eosinophil, Der f-IgE, Allergic Rhinitis, Mothers and children

## Abstract

**Objective:**

The identification of allergic rhinitis (AR) in early life is important for the target of intervention. AR is caused by various environmental factors, including house dust mites. We investigated the relationship between the *Dermatophagoides farinae* (Der f)-IgE and eosinophil in mothers with AR at delivery and the eosinophil levels and AR incidence in children.

**Methods:**

The study participants were 983 mother–child pairs from the COhort for Childhood Origin of Asthma and Allergic Diseases. AR was diagnosed by a doctor at delivery in mother and at 3 years of age in offspring. The association between eosinophil level and AR was assessed using logistic regression analysis.

**Results:**

The Der f-IgE level in mother having AR at delivery was associated with the mother’s eosinophil level, and the mother’s eosinophil level was associated with the child’s eosinophil level both at age 1 and 3. The risk of AR at age 3 in children was increased according to increased eosinophil levels in mothers at delivery and in children both aged 1 and 3 years (adjusted odds ratio [aOR] and 95% confidence interval [CI]: 2.57 [1.14–5.78], 2.28 [1.02–5.13], respectively). The risk of childhood AR at the age of 3 is increased when both mothers and children have high eosiniophils (aOR and 95% CI: 2.62 [1.01–6.79], 1.37 [0.98–1.91]).

**Conclusions:**

Der f-IgE in mothers at delivery was related to eosinophil levels in mothers with AR and higher level of eosinophils in both mother and children was associated with the increased risk of AR incidence at the first 3 years of life of children.

**Supplementary Information:**

The online version contains supplementary material available at 10.1186/s12887-023-04156-1.

## Introduction

Allergic rhinitis (AR) is a chronic respiratory disease characterized by symptoms such as rhinorrhea, nasal itching, and sneezing [1]. The prevalence of allergic diseases, such as AR, atopic dermatitis, and asthma is increasing worldwide [2]. Moreover, medical costs for treating these diseases can reach billions of dollars, which has a significant economic impact [3]. AR is the most common chronic disease in childhood [4] and is attributable to various causes, which include the maternal and prenatal environmental conditions including inflammation, dietary nutrition, psychological factors, education. These environments have important implications for fetal immune development and are associated with the development of allergic disease [5].

House dust mites (HDMs), a representative environmental factor, are the most common triggers of AR [6, 7]. The relationship between AR and HDM in childhood was confirmed through studies that identified the Skin prick tests (SPTs) positive rate of HDM in children with AR and the prevalence of AR in children sensitive to HDM [8, 9]. Moreover, in a mouse model, maternal HDM exposure during pregnancy increased the offspring’s allergic susceptibility, and vertical transmission of the maternal immune response may be involved [10]. Exposure to HDM can lead to the development of AR. The evaluation parameters for AR include the levels of IgE, basophils, and eosinophils. Among these parameters, eosinophils contribute substantially to allergic inflammation and its mechanisms [11, 12]. Increased eosinophil levels are likely to develop AR symptoms such as sneezing and nasal congestion. Studies have shown that elevated eosinophil levels are associated with childhood allergic diseases. Childhood AR reported higher eosinophil levels due to mite sensitization [13]. AR is a phenotype manifested by the interaction between genetic and environmental factors and by high eosinophilic levels and specific IgE such as *Dermatophagoides farinae* (Der f). Maternal AR along with environmental exposure is a familial risk factor for infant AR. The risk of AR is well known to have a tendency to transmit between mother and their offspring. However, there are a few studies suggesting that maternal Der f-IgE and eosinophil is associated with their offspring’s early life AR in humans.

Therefore, the present study aimed to find the relationship between Der f-IgE levels and eosinophil counts in mother with AR and the risk of AR in their offspring’s early life.

## Subjects and methods

### Study population

**Fig. 1:**
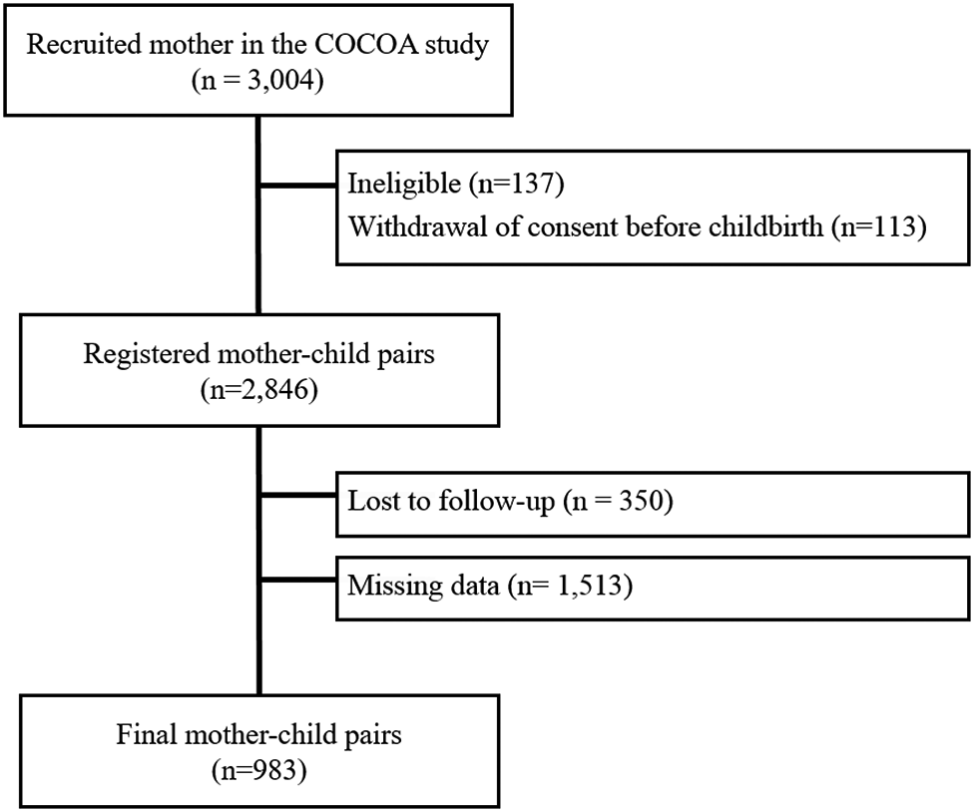
Flowchart

This study was conducted as part of the Cohort for Childhood Origin of Asthma and Allergic Disease (COCOA). COCOA is a general prospective population study as a hospital-based birth cohort designed to reveal the impact of environmental exposure in South Korea. COCOA includes demographic variables such as maternal height and weight, disease history, smoking history, tracks children’s height, environmental questionnaire responses, clinical symptoms, and disease history every year. A detailed description of the COCOA study design has been published previously [14]. A total of 3,004 pregnant women with gestational age less than 26 weeks were recruited for the COCOA study. Of these, 2,846 children were born, excluding mothers with inappropriate or withdrawn consent and those with missing follow-up. Women with missing data for major variables, such as the eosinophil level and Der f-IgE during pregnancy and the child’s sex, were excluded. Finally, 983 mother–child pairs were included in this study (Fig. 1). Written informed consent was obtained from the parents of all infants. Ethical approval for this study was obtained from the Institutional Review Board (IRB) of Asan Medical Center (IRB no. 2008–0616 and 2015–1031), Samsung Medical Center (IRB no. 2009–02–021), Severance Hospital (IRB no. 4–2008 − 0588), CHA Medical Center (IRB no. 2010–010), Seoul National University Hospital (IRB no. H-1401-086-550), and Korea Disease Control and Prevention Agency (IRB no. 2020-03-06-C-A).

### Measurement of total IgE, specific IgE, and leukocytes counts

IgE (total IgE and Der f-IgE) levels and leukocyte levels in the mother were measured at the time of the infant’s birth. IgE (total IgE, milk-specific IgE, and egg-specific IgE) levels and leukocyte levels in the children were measured at 1 and 3 years of age. Total serum IgE levels and serum levels of specific IgE to egg and milk were measured using a fluorescent enzyme immunoassay (AutoCAP System; Pharmacia Diagnostics AB, Uppsala, Sweden). Leukocytes were calculated using an automatic blood-cell counter (XE-100;Sysmex Co., Kobe, Japan) [14].

### Questionnaire data

The questionnaire based on the International Study of Asthma and Allergies in Childhood (ISAAC) was translated into Korean. The parents of infants filled out a modified version of the questionnaire. The questionnaire consists of (1) demographic factors such as gender, date of birth, height, and weight; (2) symptom history related to AR; (3) environmental factors related to allergic diseases such as residential patterns and indoor air quality [15].

### Skin prick test

SPT was performed in the mother, 6 months old infant, and in the child on the third birthday. SPT was conducted for 18 allergens (i.e. Der p, Der f, German cockroach, grasses mixture, alder, birch, oak, Japanese hop, mugwort, ragweed, dog epithelium, cat epithelium, Alternaria alternata, Aspergilus fumigatus, peanut, cow’s milk, egg white, and soybean) in children and 14 allergens (i.e. Der p, Der f, German cockroach, grasses mixture, Trees I, Trees II, mugwort, ragweed, dog epithelium, cat epithelium, Alternaria alternata, Aspergilus fumigatus, oak, and alder) in mothers using normal saline and histamine as a negative and positive control, respectively. A mean wheal size greater than 3 mm was considered a positive reaction.

### Definition of AR

AR diagnositic information was used when the child was 3 years old. AR was suspected when the participant complained symptoms of AR, such as runny nose and stuffy nose. AR was diagnosed by a physician considering SPT results (Supplementary Tables 1, 2) and the participant’s history of: (1) showed symptoms of runny nose, stuffy nose or sneezing; (2) were treated with AR; or (3) were diagnosed with AR [16].

### Statistical analyses

All statistical analyses were performed using the R program (R Foundation, Austria, available from http://www.r-project.org/foundation/main.html). Summary statistics are expressed as mean ± standard deviation (SD) or number (percentage). Two groups were compared by t-test. ANOVA was performed to determine whether maternal blood parameters differed by the maternal Der f-IgE levels at delivery. The children were divided into three groups according to the tertiles of mothers’ Der f-IgE and eosinophil levels. ANOVA was also performed to compare blood parameters in children aged 1 and 3 years according to the Der f-IgE and eosinophil levels of the mothers classified into three groups. The values divided into groups are as follows: (1) the maternal Der f-IgE: low(≤ 0.01), middle(> 0.01 to ≤ 0.92 ), and high(> 0.92); (2)maternal eosinophil: low(≤ 0.6), middle(> 0.6 to ≤ 1.2 ), and high(> 1.2); (3) infant’s 1y eosinophil: low(≤ 2.0), middle(> 2.0 to ≤ 3.5 ), and high(> 3.5); (4)infant’s 3y eosinophil: low(≤ 2.1), middle(> 2.1 to ≤ 3.7 ), and high(> 3.7). Also, logistic regression analysis was performed to determine whether maternal and childhood eosinophil counts were associated with 3-year-old AR in children. Three types of logistic regression models were constructed. The models are as follows: (i) Model 1, maternal age, BMI, total IgE at birth and secondhand smoke in pregnancy; (ii) Model 2, sex, and total IgE at 1 year old; (iii) Model 3, sex, and total IgE at 3 years of age. A logistic regression was performed to examine the association between higher eosinophilic levels in both mothers and children and the risk of AR incidence at age 3, using sex, maternal age at birth, and secondhand smoke in pregnancy as confounder variables. Significance was set at p-value < 0.05.

## Results

### Clinical characteristics of subjects

Supplementary Table 3 presents the characteristics of the study participants. The number of boys was 504 (51.3%), and there was no significant difference in the composition ratio of children by sex. The mean BMI of the mothers just before conception was 20.88 kg/m2. The mean maternal age at birth was 33.2 years. Among the mothers, 59.8% were exposed to secondhand smoke during pregnancy and 30.5% had a previous diagnosis of AR. The characteristics of COCOA subjects, including participants excluded from this study, are represented in Supplementary Table 3. There was no significant difference between the subjects regardless of the exclusion criteria for all characteristics.

### Maternal blood biochemistry parameters by maternal AR

Table 1 presents the maternal blood biochemistry characteristics of the participants according to maternal history of AR. Of the mothers, 251 (27.0%) had a history of AR. The average of Der f-IgE was 3.38 IU/mL (0,100) in mothers without AR, and 10.57 IU/mL (0,100) in mothers with AR, which was higher in mothers with AR. Eosinophils were 1.10% (0,12.9) and 1.60% (0,28.6) in the group with and without AR, respectively, and there was a significant difference between the two groups. Total serum IgE and basophils were also higher in mothers with AR. Environmental factors such as presence of animals and presence of smokers in the house were also investigated, but no significance was found.

**Table 1:** Maternal characteristics by maternal AR

	Non-AR(n = 680)		AR(n = 251)		p-value
	Number	Mean ± SD	Number	Mean ± SD	
Der f-IgE	680	3.376(± 10.815)	251	10.568(± 17.362)	< 0.001
Total IgE	676	104.04(± 299.12)	249	152.926(± 364.882)	0.059
WBC	675	8.75(± 2.862)	247	8.759(± 3.006)	0.966
Monocytes, %	680	6.916(± 1.867)	251	7.091(± 1.927)	0.214
Lymphocytes, %	680	21.475(± 8.837)	251	22.481(± 10.017)	0.162
Neutrophils, %	673	70.035(± 10.466)	246	68.508(± 11.871)	0.076
Eosinophils, %	680	1.099(± 1.162)	251	1.602(± 2.189)	0.001
Basophils, %	678	0.212(± 0.177)	249	0.254(± 0.234)	0.01

Values are presented as mean ± standard deviation

P-values are determined by t-test

### Association between Der-f IgE in mothers and blood biochemistry parameters in offsprings

Table 2 summarizes the childhood blood biochemistry according to the tertile of maternal Der f-IgE. According to the maternal Der f-IgE, there was no difference between IgE and leukocytes at 1 year of age. At 3 years of age, there was no difference according to the maternal Der f-IgE except for egg-IgE and WBC. Among maternal biochemistry parameters by maternal Derf-IgE, total IgE, eosinophils, and basophils showed significant differences and maternal eosinophils distinctly increased (Supplementary Table 4).

**Table 2:** Offspring blood biochemistry parameters by maternal Der-f IgE

	Low(n = 333)		Mid(n = 322)		High(n = 328)		p-value
	Number	Mean ± SD	Number	Mean ± SD	Number	Mean ± SD	
1y							
Total IgE	234	53.70(± 104.55)	257	69.14(± 186.99)	248	69.72(± 155.95)	0.302
Egg-IgE	234	1.05(± 3.94)	257	1.01(± 3.95)	248	1.35(± 5.72)	0.747
Milk-IgE	234	0.33(± 0.98)	257	0.28(± 0.50)	248	0.45(± 1.32)	0.159
WBC	241	8.81(± 2.55	253	8.81(± 2.65)	253	8.97(± 2.90)	0.757
Monocytes, %	241	7.99(± 2.55)	252	8.09(± 2.77)	252	8.14(± 3.39)	0.821
Lymphocytes, %	241	62.55(± 10.34)	252	62.54(± 11.20)	252	61.96(± 11.73)	0.802
Neutrophils, %	240	25.84(± 9.87)	252	25.47 ± 10.71)	251	26.10(± 10.95)	0.805
Eosinophils, %	241	2.95(± 1.77)	252	3.29 ± 2.73)	252	3.02 (± 2.07)	0.268
Basophils, %	241	0.54(± 0.40)	252	0.57(± 0.34)	252	0.57(± 0.48)	0.655
3y							
Total IgE	242	128.51(± 326.17)	201	109.88(± 215.80)	219	144.21(± 280.60)	0.362
Egg-IgE	242	1.19(± 7.63)	201	0.27(± 0.52)	219	0.98(± 4.67)	0.016
Milk-IgE	242	0.26(± 0.55	201	0.30 (± 0.63)	219	0.57(± 2.70)	0.215
Der f-IgE	238	6.02(± 19.31	200	3.54(± 12.75)	223	4.89(± 16.87)	0.257
WBC	247	6.80(± 2.33)	213	7.53(± 2.23)	230	7.24(± 2.39)	0.003
Monocytes, %	244	8.42(± 3.47)	215	7.90(± 2.68)	230	7.95(± 2.74)	0.151
Lymphocytes, %	244	52.81(± 10.77)	215	50.68(± 11.96)	230	51.77(± 10.06)	0.137
Neutrophils, %	244	34.80(± 12.20)	215	37.39(± 12.13)	229	36.29(± 11.17)	0.074
Eosinophils, %	244	3.26(± 2.26)	215	3.26(± 2.24)	230	3.39(± 2.05)	0.742
Basophils, %	244	0.66(± 0.47)	215	0.59(± 0.29)	230	0.63(± 0.46)	0.122

Values are presented as mean ± standard deviation

P-values are determined by ANOVA.

### Relationship between the maternal eosinophils and blood biochemistry parameters in offsprings

Table 3 shows the IgE and leukocyte levels in children aged 1 and 3 years in relation to maternal eosinophil levels. In the group with a low maternal eosinophil level, the eosinophil percentage in 1-year-old children was 2.80% (0.0, 20.9) and in the high group, it was 3.47% (0.6, 34.2). An increase in the maternal eosinophil level led to an increase in eosinophil levels in 1-year-old children. The eosinophil levels of 3-year-old children in the low and high groups were 2.97% (0.2, 12.6) and 3.50% (0.2, 11.3), respectively. Similarly, for children aged 3 years, when the mother’s eosinophil level increased, the child’s eosinophil level also increased, showing the same pattern as when the child was 1 year old.

**Table 3:** Offspring blood biochemistry parameters by maternal Eosinophil

	Low(n = 348)		Mid(n = 328)		High(n = 307)		p-value
	Number	Mean ± SD	Number	Mean ± SD	Number	Mean ± SD	
1y							
Total IgE	252	65.78(± 158.99)	260	57.93(± 134.88)	227	70.44(± 169.18)	0.646
Egg-IgE	252	0.85(± 2.41)	260	0.99(± 3.65)	227	1.64(± 6.88)	0.257
Milk-IgE	252	0.33(± 0.73)	260	0.28(± 0.85)	227	0.45(± 1.32)	0.223
WBC	259	8.67(± 2.67)	254	8.91(± 2.70)	234	9.03(± 2.74)	0.32
Monocytes, %	258	8.15(± 3.00)	254	7.90(± 2.68)	233	8.18(± 3.09)	0.484
Lymphocytes, %	258	61.27(± 11.75)	254	63.20(± 10.91)	233	62.61(± 10.51)	0.147
Neutrophils, %	257	27.00(± 10.67)	253	25.25(± 10.88)	233	25.09(± 9.86)	0.079
Eosinophils, %	258	2.80(± 1.99)	254	3.03(± 1.84)	233	3.47(± 2.76)	0.01
Basophils, %	258	0.56(± 0.44)	254	0.56(± 0.36)	233	0.57(± 0.43)	0.956
3y							
Total IgE	241	115.99(± 222.45)	220	143.05(± 346.54)	201	126.08(± 264.39)	0.611
Egg-IgE	241	0.94(± 7.18)	220	0.78(± 4.17)	201	0.80(± 3.71)	0.956
Milk-IgE	241	0.45(± 2.58)	220	0.36(± 0.68)	201	0.30(± 0.53)	0.412
Der f-IgE	240	4.70(± 16.72)	220	6.39(± 19.34)	201	3.47(± 13.22)	0.188
WBC	245	7.24(± 2.24)	234	7.04(± 2.40)	211	7.24(± 2.39)	0.582
Monocytes, %	244	7.97(± 2.90)	234	8.45(± 3.28)	211	7.86(± 2.77)	0.101
Lymphocytes, %	244	50.79(± 11.43)	234	52.38(± 10.59)	211	52.32(± 10.74)	0.215
Neutrophils, %	244	37.61(± 12.05)	234	34.94(± 11.48)	210	35.66(± 11.97)	0.04
Eosinophils, %	244	2.97(± 2.07)	234	3.47(± 2.29)	211	3.50(± 2.15)	0.01
Basophils, %	244	0.56(± 0.31)	234	0.71(± 0.54)	211	0.61(± 0.36)	0.001

Values are presented as mean ± standard deviation

P-values are determined by ANOVA.

### Association of blood eosinophil levels in mother and offspring and the incidence of AR in offspring at age 3

The results of the logistic regression model for the risk of developing AR at age 3 with an increasing eosinophil level are presented in Table 4. Higher eosinophils of offsprings at age 1 showed slight association with the incidence of AR (Supplementary Table 5). In the analysis of the relationship between maternal eosinophils and 3-year-old AR of children, the adjusted odds ratio (aOR) (95% CI) in the middle and high groups was 1.38 (0.73–2.62) and 1.91 (1.01–3.60), compared to the group with a low eosinophil level, respectively (Model 1). The aOR (95% CI) for the association of 1-year-old eosinophils with 3-year-old AR was 2.48 (1.11–5.58) in the middle group and 2.57 (1.14–5.78) in the high group (Model 2). Investigating the correlation between 3-year-old eosinophils and 3-year-old AR, the aORs (95% CI) were 2.13 (0.96–4.72) and 2.28 (1.02–5.13) in the middle and high groups, respectively (Model 3), implying a higher risk for AR in high group than in low group.

**Table 4:** Effect of eosinophil levels of mothers and children on the risk of childhood AR at age 3

aOR (95% CIs)	Low	Mid	High
Model 1	Ref	1.38 (0.73,2.62)	1.91 (1.01,3.60)
Model 2	Ref	2.48(1.11, 5.58)	2.57 (1.14,5.78)
Model 3	Ref	2.13(0.96, 4.72)	2.28 (1.02, 5.13)

Model 1: adjusted for maternal age at birth, maternal BMI at birth, maternal total IgE at birth, and maternal secondhand smoke in pregnancy

Model 2: adjusted for sex, and total IgE at 1y

Model 3: adjusted for sex, and total IgE at 3y

### Association between higher eosinophil levels in both mother and offspring and the risk of AR incidence

Table 5 shows the results of the logistic regression model for groups with high or low eosinophils in both mothers and children. As a result of examining the risk of 3-year-old AR when both maternal and child eosinophils were high, the aOR for maternal eosinophils was 2.62 (1.01–6.79) and the aOR for 1-year-old eosinophils was 1.37 (0.98–1.91). We confirmed that the risk of 3-year-old AR is higher when eosinophils in mother and child are higher than low.

**Table 5:** Effect of eosinophil levels on the risk of childhood AR at age 3

Variable	aOR	95% CI	p-value
Maternal eosinophils	2.62	1.01–6.79	0.048
1y eosinophil	1.37	0.98–1.91	0.064

Adjusted for sex, maternal age at birth, maternal secondhand smoke in pregnancy

## Discussion

In the present study, we identified that eosinophil levels at delivery in mothers with AR was a risk factor for AR in 3-year-old children. We confirmed high eosinophil levels in the group of mothers showing high Der f-IgE levels at delivery (Supplementary Table 4). Their children with high eosinophil levels have also been found to have an increased risk of developing AR (Table 4). These results confirm that maternal blood eosinophil according to environmental exposure is associated with childhood early onset AR.

Increased eosinophil levels in blood and bronchoalveolar lavage (BAL) when exposed to HDM for several weeks have been observed in in vivo studies [17, 18]. It is also known that there is a positive correlation between HDM-related IgE and eosinophils in children with asthma [19]. Based on these evidence, this study discovered the relationship between the maternal eosinophils and the their offsprings’ eosinophils.

Multiple studies have identified relationships between the immune system in mothers and children [20–22]. Eosinophils, IgE, and high-sensitivity C-reactive protein (hs-CRP) are factors associated with the immune system. One study identified an association between the hs-CRP level at the 24th week of gestation in the mother and that in the 6th month in the child [23] and another study identified a link between the maternal and child levels of cytokines that regulate IgE and eosinophils [24, 25]. The maternal levels of inflammatory cytokines such as interleukin-10 and TNF-α during pregnancy have been reported to be correlated with the levels of the corresponding cytokines at 1 year of age in the children [25]. In addition, a significant correlation has been reported between IgE levels in 6-month-old infants and mothers [26]. The maternal immune system is also linked to allergic diseases in the offspring [22, 25]. As such, studies have confirmed the relationship between cytokines and IgE in mothers and children, our birth cohort study showed that the higher the number of eosinophils in the mother, the higher the number of eosinophils in the young child. Here, we suggest that eosinophils, like other inflammatory indicators, show a correlation between mothers and early life children.

When an allergen, such as an HDM, enters the body, T helper 2 (Th2) cells are activated, and eosinophil levels increase [27]. This increase in eosinophils causes symptoms of AR, such as runny nose, stuffy nose, and sneezing [28]. Also, the correlation of nasal eosinophil with blood eosinophil was well known in AR patients [29] and ovalbumin-induced allergic rhinitis mouse model [30]. Severe AR is characterized by higher levels of activated eosinophils [31]. Patients with AR who were sensitive to HDMs had higher eosinophil levels than normal healthy controls [32]. Similarly, the incidence of early-onset AR can be associated with their own blood eosinophil levels, parental eosinophils, allergen induced IgE, and so on. In the present study showed that a higher incidence of AR at age 3 was observed in the both mother and offspring group with high eosinophil levels than in the group with low eosinophil levels. Also, we observed a correlation between maternal eosinophils at delivery and their offspring’s eosinophil levels both at age 1 and 3.

In addition, studies on the prevalence of AR in infants and children of various races have shown that the prevalence ranges from 6 to 32.1% [33–35]. In Korea, the prevalence of AR in infants and preschool children is 9% and 20.2%, respectively [36]. Our results for AR at 3 years of age (9.92%) were in good agreement with previous observations from several birth cohort studies. Therefore, in the present study, we could confirm the fact that the risk of AR has a tendency to transmit from mother to their infant. Also, we discovered the blood phenotype, eosinophil count, to identify infants at high risk of AR in early life. We expect that our findings are helpful for reducing the occurrence of AR in children and developing the diagnostic tool of patients in early life.

This study has several limitations that should be considered during interpretation. We obtained retrospectively the information on environmental exposures. Also, we calculated the prevalence of AR symptoms based on responses to a questionnaire. Therefore, recall bias could be occurred. However, we have many strengths. Our modified questionnaire form of ISAAC primarily concerned for responses about AR symptoms ever but less so for responses about AR symptoms in the 12 months before survey completion. Our group has validated the questionnaire in many studies and conducted the analysis of risk factors in previous studies. Also, the sample size of the present study was relatively large and the response rate was high. Moreover, we longitudinally examined every year at the same hospitals by the same researchers [15, 16, 37].

In conclusion, our study demonstrates that maternal AR at delivery is associated with the increase of Der f-IgE and eosinophil levels and maternal and their childhood eosinophil counts increased the incidence of AR in early life of offspring. Therefore, our findings for higher eosinophils in mothers and their offsprings can be helpful in the early prediction, prevention, and management of AR in childhood.

## Electronic supplementary material

Below is the link to the electronic supplementary material.

Supplementary Material 1

## Data Availability

The data are not publicly available due to it was funded by the Korea National Institute of Health and some data contained information that could compromise the privacy of research participants. However, the data used in this study are available from the corresponding author (HJ) upon request.
