# Supplementary material for: Blood eosinophil related to maternal allergic rhinitis is associated with the incidence of allergic rhinitis in offspring: COCOA study
Source: BMC Pediatr. 2023 Jul 6;23:343. doi: 10.1186/s12887-023-04156-1 (PMC10324231; doi:10.1186/s12887-023-04156-1)
Supplement: Supplementary file 1 — Supplementary Material 1 [file 12887_2023_4156_MOESM1_ESM.docx]

**Supplementary materials**

Supplementary Table 1. Maternal skin prick test by maternal AR

|  | Non-AR(n=625) | AR(n=230) | p-value |
| --- | --- | --- | --- |
| Der p | 201 (32.2%) | 156 (68.1%) | <0.001 |
| Der f | 197 (31.6%) | 149 (65.1%) | <0.001 |
| German cockroach | 33 (5.3%) | 29 (12.7%) | <0.001 |
| Grasses mixture | 15 (2.4%) | 13 (5.7%) | 0.031 |
| Trees I | 20 (3.2%) | 26 (11.5%) | <0.001 |
| Trees II | 26 (4.2%) | 30 (13.1%) | <0.001 |
| Mugwort | 35 (5.6%) | 33 (14.4%) | <0.001 |
| Ragweed | 17 (2.7%) | 20 (8.7%) | <0.001 |
| Dog epithelium | 32 (5.1%) | 42 (18.3%) | <0.001 |
| Cat epithelium | 36 (5.8%) | 44 (19.2%) | <0.001 |
| Alternaria | 10 (1.6%) | 17 (7.4%) | <0.001 |
| Aspergillus fumigatus | 12 (1.9%) | 11 (4.8%) | 0.039 |
| Oak | 18 (2.9%) | 29 (12.7%) | <0.001 |
| Alder | 13 (2.1%) | 26 (11.4%) | <0.001 |

P-values are determined by chi-squared test or fisher’s exact test.

Supplementary Table 2. Offspring skin prick test by childhood AR

|  | Non-AR(n=625) | AR(n=57) | p-value |
| --- | --- | --- | --- |
| Der p | 116 (18.6%) | 20 (35.1%) | 0.005 |
| Der f | 124 (19.8%) | 24 (42.1%) | <0.001 |
| German cockroach | 7 (1.1%) | 1 (1.8%) | 0.505 |
| Grasses mixture | 7 (1.1%) | 3 (5.3%) | 0.044 |
| Alder | 11 (1.8%) | 3 (5.3%) | 0.104 |
| Birch | 9 (1.4%) | 3 (5.3%) | 0.071 |
| Oak | 13 (2.1%) | 5 (8.8%) | 0.013 |
| Japanese hop | 7 (1.2%) | 3 (5.5%) | 0.044 |
| Mugwort | 5 (0.8%) | 2 (3.5%) | 0.110 |
| Ragweed | 3 (0.5%) | 2 (3.5%) | 0.058 |
| Dog epithelium | 20 (3.2%) | 3 (5.3%) | 0.430 |
| Cat epithelium | 9 (1.4%) | 3 (5.3%) | 0.071 |
| Alternaria | 10 (1.6%) | 2 (3.5%) | 0.265 |
| Aspergillus fumigatus | 3 (0.5%) | 1 (1.8%) | 0.295 |
| Peanut | 13 (2.1%) | 2 (3.5%) | 0.361 |
| Milk | 9 (1.4%) | 2 (3.5%) | 0.233 |
| Egg | 25 (4%) | 5 (8.8%) | 0.096 |
| Soybean | 8 (1.3%) | 2 (3.5%) | 0.200 |

P-values are determined by chi-squared test or fisher’s exact test.

Supplementary Table 3. Comparison between the characteristics of our study and COCOA study

|  | Our study | | COCOA study | |  |
| --- | --- | --- | --- | --- | --- |
|  | Number | Mean ± SD or % | Number | Mean ± SD or % | p-value |
| Infant sex (male, %) | 504/983 | 51.27 | 1,263/2,383 | 53.0 | 0.381 |
| Child birth height (cm) | 841 | 49.36 ± 2.43 | 1,912 | 49.93 ± 2.23 | 0.449 |
| Child birth weight (kg) | 873 | 31.77 ± 4.29 | 2,027 | 31.86 ± 4.27 | 0.609 |
| Gestational age (week) | 982 | 39.13 ± 1.23 | 2,285 | 39.18 ± 1.26 | 0.328 |
| Maternal age at birth (year) | 982 | 33.22 ± 3.59 | 2,297 | 33.21 ± 3.59 | 0.981 |
| Maternal body mass index(kg/m2) | 973 | 20.87 ± 2.59 | 2,339 | 20.82 ± 2.68 | 0.643 |
| Maternal educational level | 974 |  |  |  | 0.841 |
| $\leq$High school | 46 | 4.72 | 121 | 5.18 |  |
| University or college | 714 | 73.31 | 1,713 | 73.30 |  |
| Graduate school | 214 | 21.97 | 503 | 21.52 |  |
| Maternal secondhand smoke | 552/923 | 59.81 | 1,235/2,103 | 58.64 | 0.384 |
| Maternal history of allergic rhinitis | 283/928 | 30.50 | 644/2120 | 30.38 | 0.943 |

Values are presented as mean ± standard deviation.

P-values are determined by t-test.

Supplementary Table 4. Maternal blood biochemistry parameters by maternal Der-f IgE

|  | Low(n=333) | | Mid(n=322) | | High(n=328) | |  |
| --- | --- | --- | --- | --- | --- | --- | --- |
|  | Number | Mean ± SD | Number | Mean ± SD | Number | Mean ± SD | p-value |
| Total IgE | 329 | 39.63(±50.95) | 321 | 80.57(±184.34) | 326 | 225.68(±484.97) | <0.001 |
| WBC | 331 | 8.77(±2.53) | 320 | 8.73(±3.03) | 323 | 8.73(±3.25) | 0.979 |
| Monocytes, % | 333 | 7.02(±1.87) | 322 | 7.00(±1.87) | 328 | 6.84(±1.9) | 0.423 |
| Lymphocytes, % | 333 | 21.29(±8.07) | 322 | 21.93(±10.01) | 328 | 22.36(±9.89) | 0.299 |
| Neutrophils, % | 331 | 70.03(±10.13) | 319 | 69.58(±11.34) | 318 | 68.85(±11.69) | 0.393 |
| Eosinophils, % | 333 | 1.03(±1.07) | 322 | 1.16(±1.06) | 328 | 1.55(±2.15) | <0.001 |
| Basophils, % | 333 | 0.20(±0.15) | 320 | 0.24(±0.21) | 326 | 0.24(±0.22) | 0.001 |

Values are presented as mean ± standard deviation.

P-values are determined by ANOVA.

Supplementary Table 5. Offspring characteristics at age 1 by childhood AR

|  | Non-AR(n=615) | | AR(n=57) | |  |
| --- | --- | --- | --- | --- | --- |
|  | Number | Mean ± SD | Number | Mean ± SD | p-value |
| Total IgE | 609 | 66.7(±163.04) | 57 | 76.96(±146.08) | 0.617 |
| Egg-IgE | 609 | 1.25(±5.03) | 57 | 0.46(±0.90) | 0.001 |
| Milk-IgE | 609 | 0.37(±1.07) | 57 | 0.23(±0.48) | 0.06 |
| WBC | 615 | 8.76(±2.67) | 55 | 9.46(±2.81) | 0.081 |
| Monocytes, % | 613 | 8.13(±3.01) | 56 | 8.09(±2.77) | 0.92 |
| Lymphocytes, % | 613 | 62.52(±11.00) | 56 | 61.18(±11.09) | 0.389 |
| Neutrophils, % | 612 | 25.65(±10.35) | 56 | 25.99(±10.13) | 0.808 |
| Eosinophils, % | 613 | 3.01(±1.96) | 56 | 4.07(±4.44) | 0.082 |
| Basophils, % | 613 | 0.56(±0.39) | 56 | 0.59(±0.39) | 0.521 |

Values are presented as mean ± standard deviation.

P-values are determined by t-test.
